# Proteomic profiling of the neurons in mice with depressive-like behavior induced by corticosterone and the regulation of berberine: pivotal sites of oxidative phosphorylation

**DOI:** 10.1186/s13041-019-0518-4

**Published:** 2019-12-30

**Authors:** Qin Gong, Xiao-Jin Yan, Fan Lei, Mu-Lan Wang, Lu-Ling He, Ying-Ying Luo, Hong-Wei Gao, Yu-Lin Feng, Shi-Lin Yang, Jun Li, Li-Jun Du

**Affiliations:** 1 Jiangxi University of Traditional Chinese Medicine, Nanchang, 330006 China; 2 State Key Laboratory of Innovative Drugs and Efficient Energy-saving Pharmaceutical Equipment, Jiangxi University of Traditional Chinese Medicine, Nanchang, 330006 China; 3 School of Life Sciences, Tsinghua University, Beijing, 100084 China; 4 College of Pharmacy, Guangxi University of Chinese Medicine, Nanning, 530000 China

**Keywords:** Corticosterone, Proteomic analysis, Mitochondria, Depression, Berberine

## Abstract

Chronic corticosterone (CORT) stress is an anxiety and depression inducing factor that involves the dysfunction of glucocorticoid receptor (GR), brain-derived neurotrophic factor (BDNF), and neuronal plasticity. However, the regulation of proteomic profiles in neurons suffering CORT stress is remaining elusive. Thus, the proteomic profiles of mouse neuronal C17.2 stem cells were comprehensively investigated by TMT (tandem mass tag)-labeling quantitative proteomics. The quantitative proteomics conjugated gene ontology analysis revealed the inhibitory effect of CORT on the expression of mitochondrial oxidative phosphorylation-related proteins, which can be antagonized by berberine (BBR) treatment. In addition, animal studies showed that changes in mitochondria by CORT can affect neuropsychiatric activities and disturb the physiological functions of neurons via disordering mitochondrial oxidative phosphorylation. Thus, the mitochondrial energy metabolism can be considered as one of the major mechanism underlying CORT-mediated depression. Since CORT is important for depression after traumatic stress disorder, our study will shed light on the prevention and treatment of depression as well as posttraumatic stress disorder (PTSD).

## Introduction

Depression is a common mental disorder worldwide [1]. The pathogenesis of depression is not completely clear [2]. In addition to serotonin (5-HT) knowledge, immune activation and the production of inflammatory cytokines, such as IL-1β, IL-6, TNFα, are involved in depression because its symptomatology includes some behaviors that also occur during chronic inflammatory stress [3–6]. Recent studies suggest that the beneficial effect of antidepressant drugs is mediated via stimulation of adult hippocampal neurogenesis and subsequent increase in hippocampal plasticity. Changes in hippocampal neurons can play an important role in the pathogenesis of depression, involving the possible mechanism of ERK (extracellular signal-regulated kinase) pathway [7], AMPK (Adenosine monophosphate- activated protein kinase) pathway [8], GABAergic dysfunction in the nucleus accumbens (NAc) [9], epigenetic events altering the chromatin structure and thus modulating expression of genes [10], Sirt1 (silent information regulator 1) at the consumption of NAD+ [11], BDNF pathway [12,13], etc. However, mitochondrial energy metabolism in depression remains poorly understood although the action of the above mentioned signalings, at least in part, relies on energy metabolism.

Glucocorticoid (GC) disorder, another important factor that induces depression, often occurred in chronic depression [14,15]. GC binding to the intracellular glucocorticoid receptor (GR) stimulates the translocation of the GR from the cytosol to the nucleus, leading to the transactivation or transrepression of gene transcription [16]. Thus, GR in neuron plays key roles in the stress system by maintaining molecular, cellular and systemic homeostasis in neurons via the HPA (hypothalamic-pituitary-adrenal) axis [17]. Dysfunctions in the GR had been implicated in the pathogenesis of depression [18]. Chronic high concentrations of GC, associated with down-regulated BDNF expression in hippocampus, can cause anxiety and depression-like behavior, which in turn affects neuronal plasticity, learning and memory, and severe neuronal injury [19–22].

Although the long-term injection of CORT is widely used to induce depressive-like behavior in mice [23,24], the mechanism underlying CORT regulating neuron functions is not yet fully studied. Comprehensive studies in the effects of CORT on neurons in the aspect of proteomics could be beneficial for a deeper and systematic understanding of the effects of CORT-regulated brain function, as well as the application of CORT-treatment in future experimental research.

Berberine (BBR) is a natural small molecule widely used in clinics. Our published works described the details process regarding the distribution of BBR in the neurons in hippocampus after intravenously injecting BBR to mice [25–28]. BBR executes neuroprotective activities via showing anti-inflammatory and anti-apoptotic effects, such as IL-1β, TNF-α, NFκB, Caspase3, etc. [29]. Recent studies have discovered BBR’s effect on the other signaling pathways, such as PI3K-Akt, p38MAPK and AMPK signaling etc., in brain disorder [30–32], showing the complex mechanism of BBR neuroprotective effect. The anti-depression and anti-anxiety effects are newly identified pharmacological activities of BBR against neuropsychiatric disorders [33–37]. However, the mechanism of the well evidenced efficacy is remaining unclear. Thus, the underlying mechanism of BBR effect on the CORT induced depression needs to be studied deeply.

As depression is not just a neurotransmitter disorder in synapses, it is mainly related to the function disorder of hippocampal neurons. This abnormality in neuronal function is manifested by a decrease in neuron plasticity which is depended on the energy supplement from mitochondria in a large extent. Therefore, we hypothesized that (1) there must be a complex proteomic profile in neural system during CORT-induced depression; (2) mitochondrial energy metabolism in hippocampal neuron could be involved in the mechanism underlying this depression; (3) BBR could have effects on the proteins correlating to the energy metabolism in this pathphysiological process.

In the present study, we investigated the proteomic profiles of mouse neural stem cells in vitro and then confirmed the proteomic changes in the mouse brain based on the depressive-like behavior model that induced by CORT*,* to comprehensively understand the proteomic alterations in CORT-induced depression. The results revealed that mitochondrial energy metabolism disorder is a novel mechanism underlying CORT-induced depression, and BBR executed anti-depression effects via antagonizing the proteomic disorders, which in turn as the behavior disorders, that induced by CORT treatment.

## Materials and methods

### Cells and animals

C17.2 cells, a gift from Dr. Wei-Dong Xie of Shenzhen Graduate School at Tsinghua University, are a prototypical and stable neural stem cell (NSC) line that is valuable for in vitro studies in understanding neural cell activity [38,39]. Dulbecco’s modified Eagle’s medium (DMEM) was obtained from Gibco (New York, USA). BBR was obtained from Beijing Shuanghe Pharmacy (Beijing, China), and CORT was purchased from Sigma-Aldrich (Shanghai, China).

Male C57BL/6 mice, weighing 18–20 g, were purchased from Beijing Vital River Laboratory Animal Technology Co., Ltd. (Beijing, China). This experiment was carried out at the Laboratory of Barrier Environment of the Jiangxi Bencao-Tiangong Technology Co., Ltd. (Nanchang, China). The animals were housed in temperature- and humidity-controlled rooms under a 12-h light/dark cycle and provided with unrestricted amounts of rodent chow and drinkable water. All procedures described were reviewed and approved by the Institutional Animal Care and Use Committee of Jiangxi University of Traditional Chinese Medicine and the Animal Welfare and Ethics Committee of Jiangxi University of TCM (approval ID: 19-JunLi-CORT). The experimental procedure strictly followed the guidelines of the Experimental Animal Welfare and Ethics of China.

### MTT assay for cell viability

The MTT (3-(4,5-dimethylthiazol-2-yl)-2,5-diphenyltetrazolium bromide) assay was performed as previously described [40]. Briefly, the cells were seeded onto 96-well plates and cultured for 12 h, followed by treatment with the indicated dose of BBR or CORT for 24 h. Subsequently, MTT solution was added at a final concentration of 0.5 mg/ml, and the cells were incubated for 4 h. Then, the medium was removed, and 0.1 ml DMSO (dimethyl sulfoxide) was added to each well. The absorbance at 550 nm was measured using a microplate reader (Bio-Rad, USA.), and the viability (%) was determined by comparison with the control group.

### Protein preparation, digestion and TMT labeling

For the protein preparation, C17.2 cells were seeded onto 10-cm plates at an adequate concentration, cultured overnight. The samples from the four groups, normal control group (saline), CORT (100 μmol/L) group, CORT (100 μmol/L) + BBR (1.5 μmol/L) group and normal control + BBR(1.5 μmol/L) group. Subsequently, the cells were harvested and lysed using lysis buffer (Beyotime, China). The cell lysates were centrifuged (12,000 g, 10 min, 4 °C), and the supernatants were collected. The protein concentration was determined using a BCA Protein Assay Kit (Beyotime, China). A total of 20 μg of protein from each group was separated by 10% SDS-PAGE, and the gel was subsequently stained with Coomassie Brilliant Blue R-250.

For protein digestion, the entire gel was cut into pieces, and the excised gel pieces were destained and dried using 25 mmol/L NH_4_HCO_3_ containing 50% acetonitrile. Subsequently, the gel pieces were successively incubated in 50 mmol/L NH_4_HCO_3_ containing 25 mmol/L dithiothreitol (DTT) and 50 mmol/L NH_4_HCO_3_ containing 55 mM iodoacetamide (IAA), followed by washing with 100 mmol/L NH_4_HCO_3_ and drying overnight. The gel pieces were digested using sequencing grade modified trypsin in 50 mmol/L NH_4_HCO_3_ at 37 °C overnight. The digested peptides were extracted twice with 50% acetonitrile aqueous solution containing 0.1% trifluoroacetic acid.

For tandem mass tag (TMT) labeling, the extracted peptides were enriched and re-dissolved in 200 mmol/L tetraethylammonium bromide (TEAB), and TMTsixplex Label Reagent (Thermo Scientific, USA) was added to each sample according to the manufacturer’s instructions. The reaction was incubated for 1 h at room temperature, and 8 μl of 5% hydroxylamine was subsequently added to the sample and incubated for an additional 15 min to quench the reaction.

### Detection of C17.2 cell proteins using LC-MS/MS

Equal amounts of labeled peptides from the three samples were combined and analyzed by HPLC (high-performance liquid chromatography) analysis. The TMT-labeled peptides were separated by gradient elution at a flow rate of 0.3 μl/min for 120 min by using a Thermo-Dionex Ultimate 3000 HPLC system. The analytical column contained 300 Å C-18 resin (Upchurch, USA) packed into a fused silica capillary column (75 m ID, 150 mm length; Varian, USA). Mobile phase A comprised 0.1% formic acid, and mobile phase B comprised 80% acetonitrile and 0.08% formic acid. The gradient was 0 min-4% B, 5 min-4% B, 85 min-35% B, 95 min-50% B, 100 min-95% B, 105 min-95% B, 110 min-4% B, and 120 min-4% B.

Mass spectrometry (MS) analysis was performed with a Thermo Scientific Q Exactive mass spectrometer operated using Xcalibur 2.1.2 software in data-dependent acquisition mode. A single full-scan mass spectrum in the orbitrap (400–1800 m/z, 60,000 resolution) was performed, followed by 10 data-dependent MS/MS scans at 30% normalized collision energy (NCE). The spray voltage was 2.3 kV.

For protein identification, the MS/MS spectra from each LC-MS/MS run were searched against the mouse FASTA from UniProt using Proteome Discoverer software (Thermo Scientific, USA). The search criteria were as follows: full tryptic specificity was required; one missed cleavage was allowed; carbamidomethylation (C) and TMTsixplex (K and N-terminal) were set as the fixed modifications; oxidation (M) was set as the variable modification; and precursor ion mass tolerances were set at 10 ppm for all MS acquired with the orbitrap mass analyzer. The peptide false discovery rate was calculated using Percolator software. Only peptides assigned to a given protein group were considered unique. A peptide spectrum match with a q value threshold of 1% was considered. The peptide spectrum matching the reverse, decoy database was considered false discovery, and the false discovery rate was set to 1% for protein identification.

Relative protein quantification was performed using Proteome Discoverer software according to the manufacturer’s instructions. Quantitation was only conducted for proteins with at least two unique peptide matches. The average of all peptide hits to a protein served as ratios, and the variability served as the quantitative precision.

### Gene ontology

Gene Ontology (GO) analysis was performed using the DAVID database (http://david.abcc.ncifcrf.gov/) following a previously established protocol [41,42]. The proteins were annotated based on molecular function, biological process, and cellular components. The Kyoto Encyclopedia of Genes and Genomes (KEGG) database (http://www.kegg.jp/) was used for the signaling pathway analysis. A threshold of count > 2 and EASE < 0.05 (*P* < 0.05) was set in the annotation analysis. The signaling networks were carried out using the String Database: Functional protein associated networks (https://string-db.org/) [43].

### Mouse model with depressive-like behaviors induced by CORT

The mice were randomly divided into four groups of 6–9 mice in each group: normal control group, CORT group, CORT + BBR group and normal control + BBR group. The experimental course lasted 3 weeks. In the first week (day 0 to day 7), the mice were injected with CORT by subcutaneous injection (s.c.) (dosage of 20 mg/kg per day) [44]. From week 2 to week 3 (day8 to day20), BBR was administered at a dose of 150 mg/kg by oral administration (equivalent to the adult dosage in the clinic), and CORT was continuously injected simultaneously. Saline was given in the normal control. On day 20, all mice exhibited depressive-like behavior in the manner of sucrose preference according to the reference [45, 33]. The quantity of sucrose water and urine produced in 24 h for each mouse was determined by using an Y3101 mouse metabolism cage (Shanghai Yuyan Instruments Co., Ltd., Shanghai, China). After the detection of sucrose water and urine, the mice were anesthetized with 10% urethane, and the brains were isolated and stored at − 80 °C.

### The expression of mRNA and protein

The expression of mRNA was carried out using real-time PCR assay according to the reference [46]. Total RNA was extracted from mouse hippocampus using an RNA extraction kit (Tiangen Biotech, China) and reverse-transcribed to cDNA using the Fastquant RT Kit (Tiangen Biotech, China) according to the manufacturer’s instructions. Real-time PCR for specific genes was performed on a 7500 Real Time PCR system (Applied Biosystems, USA) using a SYBR Green Master Mix kit (Tiangen Biotech, China) according to the manufacturer’s instructions. *β*-actin was used as an internal control. The primers were designed with GenBank NCBI (https://www.ncbi.nlm.nih.gov/) and manufactured by Shenggong Biotech Company (Shanghua, China) (Table 1).

**Table 1:** The primers used in the study

Name	Sense	Antisense
*β*-actin	5′- CCACTGTCGAGTCGCGT − 3′	5′- CCCACGATGGAGGGGAATAC − 3′
Ndufb11	5′-TAAGGGCGGAGCGACAAAAA − 3′	5′- GACAGGCAGCGACCATACAA − 3′
Ndufb4	5′- GTGCGCCGGGAGTCAAG − 3′	5′- CCAGCGAATCAAGGCAGGAT − 3′
Ndufb5	5′-CTGGAGGTCTGGGAAGTTGTG-3′	5′-AGTCCCAAATAAGCCCCATCTG-3′
Ndufb6	5′- TTTAAGGCGTACCGCTCCAG −3′	5′- TCCTGGGCTTCGAGCTAACA − 3′
Ndufs4	5′- GGCGGTCTCAATGTCAGTGT −3′	5′- TGTCCCGAGTCTGGTTGTCT − 3’
Ndufa12	5′- ACGGGTTTTCTTCAGGGCAA −3’	5′- GCACCATGCTTCCATCCACA − 3’
Ndufaf2	5′- CCGAGTGGGAAGCATGGATT −3’	5′- TCCCTTCCAAAATACGGGGC − 3’
Ndufa6	5′- AGTATGGAAGCAGCGGACAC −3’	5′- ATGCACCTTCCCATCAGGTG − 3’
Ndufa7	5′- CCGCTACTCGCGTTATCCAA −3’	5′- TTGGACAGCTTGTGACTGGG − 3’
Ndufa8	5′- TCGCCCTTTGCCAGAGAATC −3’	5′- ACCGTCGACCCATCTCTACA − 3’
Mrps10	5′-CTGCAGCAGGAATTGGGGAAT-3’	5′- GTGGCACCCACTTCATACTGG −3’
Mrps17	5′- ATACTCCCTAACCCGGGACC −3’	5′- CACCTTCCCCACAACCCATT −3’
Mrps6	5′- AATCCCTGATGGACCGAGGA −3’	5′- TTCTCCACAGCACTTGTCGG −3’
Uqcc2	5′- ACCGGCGTTTCCTTAAGCTCT −3’	5′-GCTAAGCTCTCGTACATCTGGG-3’
BDNF	5′- GTAAACGTCCACGGACAAGG −3’	5′- ATGTCGTCGTCAGACCTCTC −3’
CREB	5′- AGCCTCAGCACGATACCTAC −3’	5′- CTCCGTAGGTCCTGAGTCAC −3’
GR	5′- GGTGGAGCTACAGTCAAGGT −3’	5′- TGCTTGGAATCTGCCTGAGA −3’
AMPA	5′- GGATGGCTCTGAGGTCATGT −3’	5′- GCAGGTAGAAGGCGAGTTTG −3’
NMDA	5′- TCAGTCTGAGCAGTGGAAGG −3’	5′- CGAGGGTAAGGAGACATCCC −3’

Protein expression was analyzed using Western blotting as previously described [47]. For the analysis, primary antibodies were shown in Table 2. The goat anti-mouse IgG-HRP (ZB2305) and goat anti-rabbit (ZB2301) IgG-HRP secondary antibodies were purchased from ZSGB-Bio (Shanghai, China) and used with dilution of 1:2000. The targeted proteins were visualized with the Super Signal West Femto Chemiluminescent Substrate (Thermo Scientific Pierce, Beijing, China), and the intensities of the visualized bands were analyzed using Quantity One software (Bio-Rad, Shanghai, China). *β*-actin was used as an internal control. The data were expressed as the ratio to *β*-actin.

**Table 2:** The primary antibodies used for the protein expression in the study

Name	Resource	Dilution	Match number and company
BDNF	Rabbit polyclonal antibody	1:1000	28,205–1-AP, Proteintech at Shanghai, China
CREB	Rabbit monoclonal antibody	1:1000	ab32515, Abcam at Shanghai, China
GR (Ab211)	Rabbit polyclonal antibody	1:1000	TA313373, Origene at Shanghai, China
AMPA	Rabbit monoclonal antibody	1:1000	ab109450, Abcam at Shanghai, China
NMDA	Rabbit polyclonal antibody	1:1000	A327881, Origene at Shanghai, China
*β*-actin	Mouse monoclonal antibody	1:1000	TA-09, Zhongshan Jinqiao Biotech Company, Beijing, China

### Data analysis

The data were expressed as the means ± S.E.M. The data were statistically analyzed using a one-way ANOVA for the F test and then a *t*-test between groups. The chart data were processed using GraphPad Prism 8.01 software (GraphPad Company, San Diego, California, USA). A *P* < 0.05 was considered statistically significant. The proteomic analysis was performed using the DAVID Bioinformatics Resources 6.8 database (https://david.ncifcrf.gov/), and protein networks were carried out using String: Functional Protein Associated Networks (https://string-db.org/).

## Results

### Cell viability with BBR or CORT administration

**Fig. 1:**
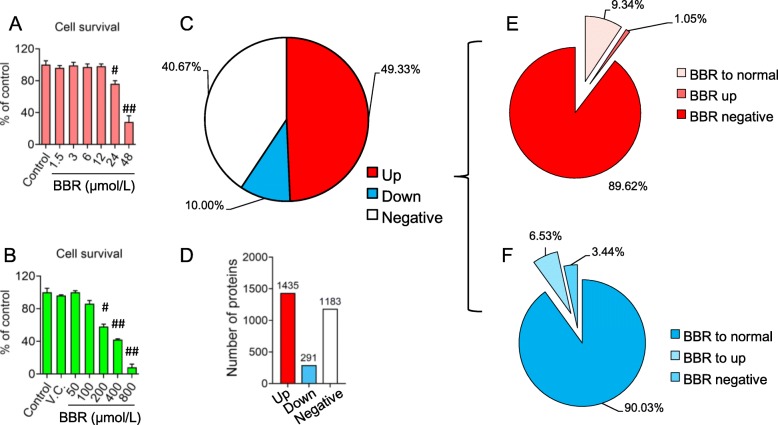
Distribution of proteins of C17.2 cells detected by LC/MS-MS. **a** - **b** Viability of c17.2 cells after berberine (BBR) and corticosterone (CORT) treatment using MTT assay. The data are shown as the means ± S.E.M from 6 independent experiments (*n* = 6). # vs. control groups, *P* < 0.05. ## vs. control groups, *P* < 0.01. VC: vehicle control. **c** - **d** General statistics of protein detected after CORT administration. Up: up-regulation of protein compared with normal control. Down: down-regulation of protein compared with normal groups. Negative means there were no obvious change of protein level compared with normal groups. The number on each bar of **d** indicates the number of proteins detected. **e** Effect of BBR on the up-regulated proteins induced by CORT. BBR to normal: BBR suppressed the upregulated protein to normal level compared with normal control. BBR to up: BBR acted the upregulated proteins but also in high level. BBR negative: No effect of BBR on the upregulated protein induced by CORT. **f** Effect of BBR on the down-regulated proteins induced by CORT. BBR to normal: BBR enhanced the down-regulated protein to normal level compared with normal control. BBR to up: BBR acted the down-regulated proteins in high level. BBR negative: No effect of BBR on the down-regulated protein induced by CORT

The cytotoxicity of BBR or CORT treatment was firstly studied by using MTT assay. The highest tolerant concentration of BBR in vitro was 12 μmol/L (Fig. 1a). Due to the limited bioavailability of BBR via oral administration to animal, the peak concentration of BBR in the blood could not be higher than 1.5 μmol/L (0.5 μg/ml), which is commonly used in pharmacological research [48]. Therefore, the dosage of 1.5 μmol/L was used for the proteomics study. Given to the 24 h-treatment of 200 μmol/L CORT decreased cell viability significantly (Fig. 1b), a cytotoxicity-free dosage of CORT (100 μmol/L) was used for cell modeling [49].

### Protein identification and quantification

The TMT-labeling quantitative proteomics analysis was applied to investigate the protein expression profiles of BBR- or CORT-treated C17.2 cells. Protein samples from the control, BBR and CORT groups were separated through SDS-PAGE, and the gel was cut into seven segments. After gel dissolution and digestion, were labeled. The proteomic studies confidently identified 2909 proteins in the samples. CORT treatment upregulated (2.3 folds to the corresponding proteins in normal control group) the expression of 1435 proteins (49.33% of the identified proteins), while 291 proteins (10% of the identified proteins) were downregulated (the abundance is less than 0.7 compared with the control group). The expression of 1183 proteins (40.67% of the identified proteins) showed no significant change in the CORT treatment (Fig. 1c and d). BBR treatment antagonized the upregulation of 134 proteins, which accounts for the 9.34% of 1435 proteins that upregulated by CORT treatment. Additionally, BBR treatment further stimulated the expression of 15 proteins (1.05%) that were upregulated in CORT treatment, revealing a synergistic effect of CORT and BBR treatment on the selected proteins (Fig. 1e). Notably, BBR upregulated the expression of 29 proteins (9.79% of 291 proteins) that were down-regulated by CORT treatment (Fig. 1f), indicating the antagonistic effect of BBR on the CORT-mediated protein expression.

### Annotation of proteomic function in general

**Fig. 2:**
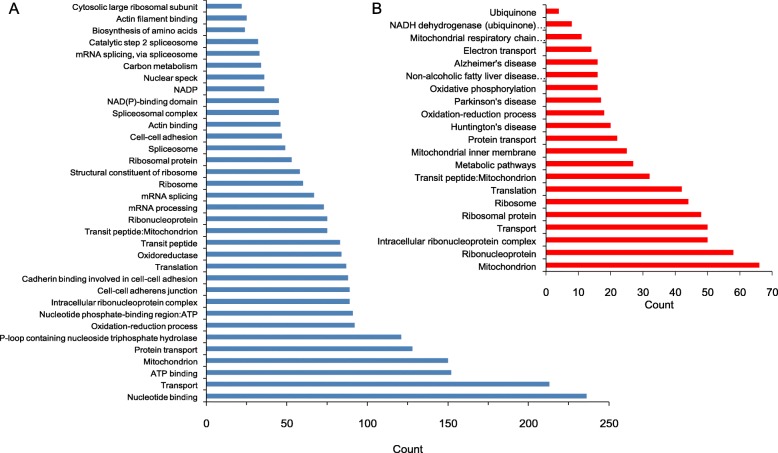
Annotation clustering of proteins regulated after the administration of CORT in C17.2 cell in vitro. **a** The clustering of proteins down-regulated after CORT. **b** The clustering of proteins up-regulated after CORT

The identified proteins were analyzed by using the DAVID Bioinformatics Resources 6.8 database (https://david.ncifcrf.gov/). The CORT down-regulated proteins involve variant cellular biological processes, such as ribosomal protein, translation, transit peptide, mitochondrial oxidation-reduction process, protein transport, ATP binding, etc. (Fig. 2a). The biological processes annotation of mitochondria and the oxidation-reduction process suggest that the neuronal changes caused by CORT involve mitochondrial function. Given to the pivotal function of mitochondria in energy metabolism, the mitochondrial energy metabolism changes in the neural cell might be the mechanism underlying CORT-induced depression (Fig. 2b).

### Function annotation of CORT up-regulated proteins

**Fig. 3:**
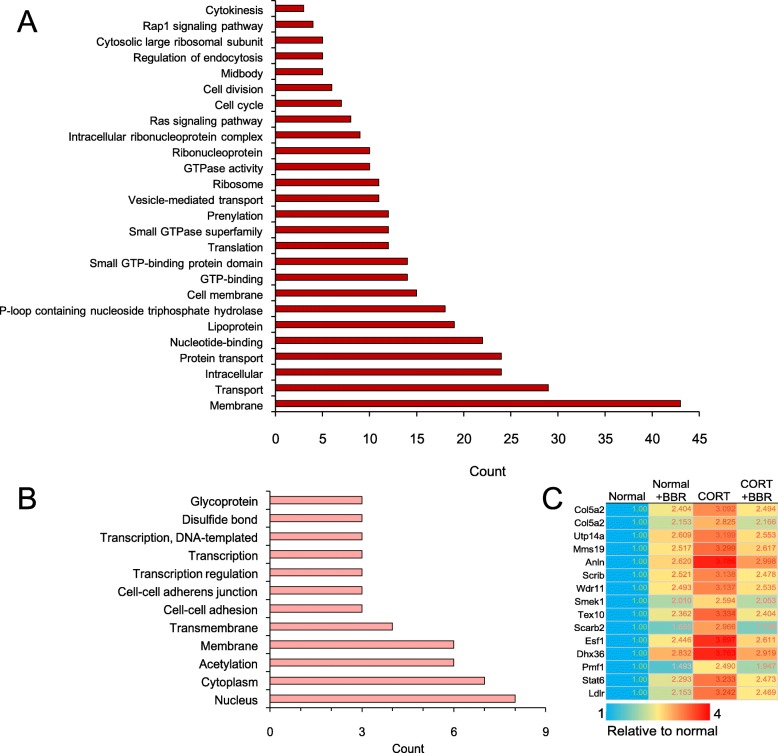
Annotation clustering of the proteins up-regulated by corticosterone (CORT) and alternated by berberine (BBR) in C17.2 cells in vitro. **a** Proteins up-regulated by CORT but BBR suppressed to normal level. **b** Function clustering of the proteins up-regulated by CORT but BBR had little effect. **c** Genes of the protein up-regulated by CORT but BBR had little effect

Among the proteins that upregulated by CORT, BBR can antagonize about 9.79% of CORT-mediated protein upregulation. The function annotations of the involved proteins are the nucleus, cytoplasm, cell membrane, GTP binding, cell cycle, cell division, cytokines, etc. (Fig. 3a). There were another 15 proteins weakly suppressed by BBR, including glycoprotein, cytoplasm, transmembrane, cell-cell adhesion, and transcription regulation (Fig. 3b). These upregulated proteins by CORT, compared to the annotation of mitochondrial energy metabolism, are poorly related with a specific functional annotation (Fig. 3c), further strengthen the hypothesis that CORT induces depression via suppressing mitochondrial energy metabolism in the neural cell.

### Function annotation of CORT down-regulated proteins

**Fig. 4:**
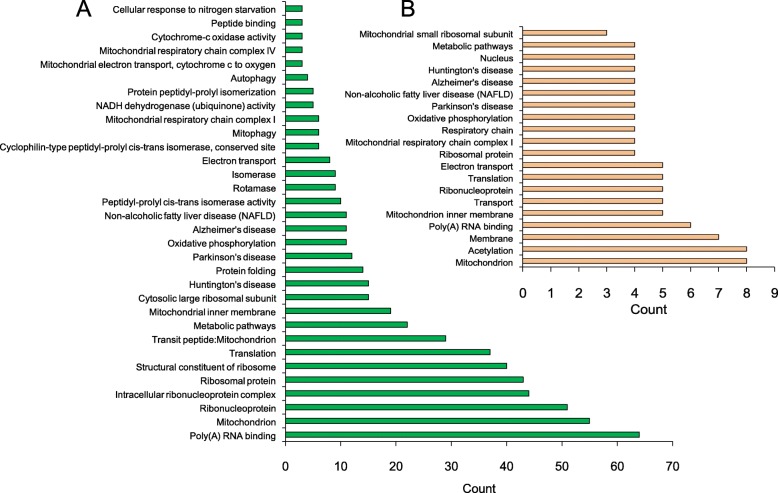
Annotation clustering of proteins down-regulated after the administration of CORT and BBR activation in C17.2 cell in vitro. **a** Proteins down-regulated by CORT but BBR promoted to normal level. **b** Functions of the proteins down-regulated by CORT but BBR enhanced in high level

Quantitative proteomics analysis revealed that BBR upregulated the CORT-down regulated proteins, which involves various cell functions and processes, such as ribonucleoprotein, poly(A) RNA binding, translation, mitochondrion, protein folding, protein transport, oxidative phosphorylation, electron transport, respiratory chain, NADH dehydrogenase (ubiquinone) activity, metabolic pathways, autophagosome assembly, etc. (Fig. 4a). Moreover, BBR can significantly upregulate the expression of CORT treatment-suppressed 14 proteins, which involves the functions of mitochondrion, oxidative phosphorylation, electron transport, respiratory chain, NADH dehydrogenase (ubiquinone) activity, metabolic pathways, and mitochondrial respiratory chain complex I (Fig. 4b).

**Fig. 5:**
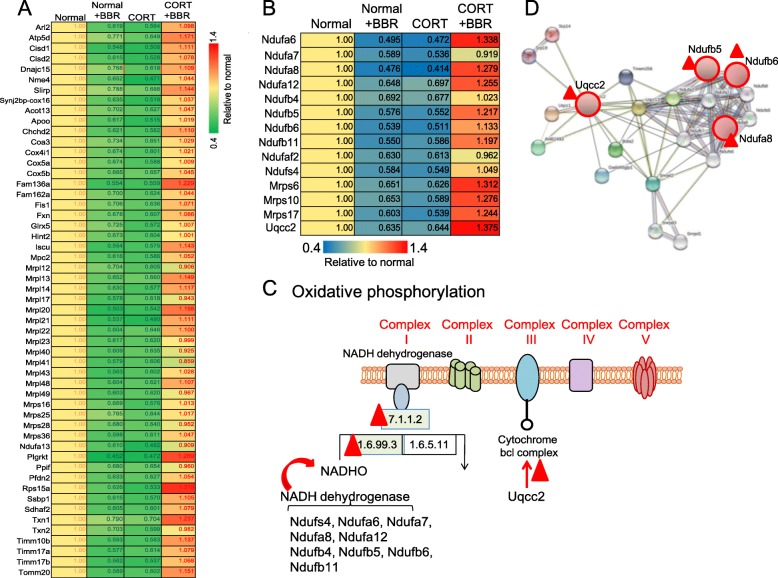
The variation of protein expression after administration of corticosterone (CORT) and berberine (BBR) indicating the correlation to mitochondrion. **a** Genes of the proteins detected suppressed by CORT but reversed to normal level by BBR compared with normal control. **b** Genes of the proteins suppressed by CORT but increased in higher level distinctly by BBR compared with normal control. **c** Schematic of the sites of BBR for oxidative phosphorylation in mitochondria respiratory chain based on the Functional Annotation Chart of David Bioinformatics Resources 6.8 (https://david.ncifcrf.gov/). **d** Networks of Uqcc2 to NADH dehydrogenases based on String: Functional protein associated networks (https://string-db.org/). “Red triangle”: Target of BBR action

Further analysis revealed that many of the downregulated proteins caused by CORT are involved in the mitochondrial respiratory chain. BBR also has a down-regulation effect on normal cells, but when used with CORT-treated cells (Fig. 5a). Some of the affected proteins are relevant with NADH dehydrogenase, *Ndufb5*, *Ndufb6*, *Ndufa7* and ubiquinol-cytochrome c reductase complex *Uqcc2* of mitochondria (Fig. 5b and c) have network relation (Fig. 5d) based on the results of the David Bioinformatics Resources database (https://david.ncifcrf.gov/) (Table 3), suggesting the multi-targets and synergic effect of BBR on the CORT induced depressive disorder.

**Table 3:** Gene name and the description based on the David Bioinformatics resources database

Accession	Gene abbreviation	Description
Q9CQZ5	Ndufa6	NADH dehydrogenase (ubiquinone) 1 alpha subcomplex, 6 (B14)
Q9Z1P6	Ndufa7	NADH dehydrogenase (ubiquinone) 1 alpha subcomplex, 7 (B14.5a)
Q9DCJ5	Ndufa8	NADH dehydrogenase (ubiquinone) 1 alpha subcomplex, 8
Q7TMF3	Ndufa12	NADH dehydrogenase (ubiquinone) 1 alpha subcomplex, 12
Q9CQC7	Ndufb4	NADH dehydrogenase (ubiquinone) 1 beta subcomplex 4
Q9CQH3	Ndufb5	NADH dehydrogenase (ubiquinone) 1 beta subcomplex, 5
A2AP32	Ndufb6	NADH dehydrogenase (ubiquinone) 1 beta subcomplex, 6
O09111	Ndufb11	NADH dehydrogenase (ubiquinone) 1 beta subcomplex, 11
Q59J78	Ndufaf2	NADH dehydrogenase (ubiquinone) 1 alpha subcomplex, assembly factor 2
E9QPX3	Ndufs4	NADH dehydrogenase (ubiquinone) Fe-S protein 4
P58064	Mrps6	Mitochondrial ribosomal protein S6
E9QJS0	Mrps10	Mitochondrial ribosomal protein S10
D3Z198	Mrps17	Mitochondrial ribosomal protein S17
D3Z4C9	Uqcc2	Ubiquinol-cytochrome c reductase complex assembly factor 2

### The validation of protein expression in a mouse model of depression induced by CORT

To further determine the proteomic results of C17.2 cells, we studied the genes related to the oxidative respiratory chain in the hippocampus of mouse with or without CORT treatment. In addition to the genes involved in energy metabolism, the markers that indicate the neural changes induced by CORT, such as BDNF, GR, CREB, AMPA (α-amino-3- hydroxy-5- methyl-4-isoxazolepropionic acid) and NMDA (N-methyl -d-aspartic acid) receptors were studied by using Real-Time PCR.

**Fig. 6:**
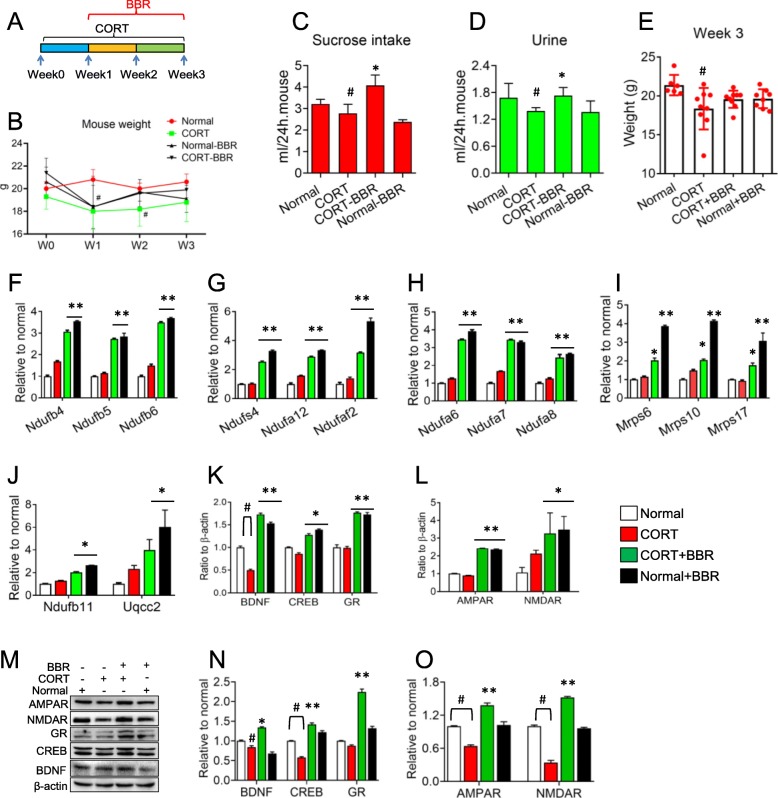
mRNA expression of the proteins in mouse brain after chronic injection of corticosterone (CORT). **a** Schedule of the experiment. **b** Body weight of mice. **c** Sucrose intake of mice at the end of the experiment. **d** Quantity of mouse urine. **e** Body weight at the end of the experiment. **f** - **j** mRNA expressions of the proteins detected by LC/MS. **k** - **l** mRNA expression of the markers reflecting neural disorder after CORT administration. **m** - **o** Protein expression of the markers. W: week. BBR: berberine. The data were shown as mean ± S.E.M from eight to nine mice in each group. # vs. the normal control, *P* < 0.05. *, ** vs. CORT groups, *P* < 0.05, *P* < 0.01. NS means no significance

After 21 days of CORT injection, the amount of sucrose water consumed by the mice was significantly reduced ((2.78 ± 0.42) ml / 24 h per mouse) compared with normal groups ((3.22 ± 0.21) ml / 24 h per mouse) (*P* < 0.05), and the quantity of urine was also significantly decreased ((1.39 ± 0.07) ml / 24 h per mouse) compared with the normal ((1.68 ± 0.32) ml / 24 h per mouse) (*P* < 0.05). In the group of mice treated with BBR for 14 days, the water consumption increased significantly ((4.08 ± 0.21) ml / 24 h per mouse) and the urine volume increased obviously ((1.73 ± 0.18) ml / 24 h per mouse) compared with CORT groups (*P* < 0.05), indicating that the depressive-like behaviors of mice were improved. Simultaneously, the weight of the mice in the CORT group was at (18.4 ± 2.7) g and significantly lower than that in the normal group (21.4 ± 1.3) g (*P* < 0.05), while the body weight in the groups with BBR was not significantly reduced compared with normal mice although it looks a reduced tendency compared with the normal (*P* = 0.144) (Fig. 6a-e). In Fig. 6c and d, BBR showed the suppression of sucrose intake and urine discharge on normal mice. However, BBR could reverse the reduction of sucrose intake and urine volume caused by CORT. This effect characterized by suppression in normal conditions but increase in pathophysiological conditions shows the complex of BBR action, which needs in depth-study further.

BBR can upregulate mRNA expressions of oxidative phosphorylation of mitochondria in the CORT group and even in normal mice, which is in agreement with the proteomic detection results. In the expression of *Ndufb4*, *Ndufb5*, *Ndufb6*, *Ndufa6* and *Ndufa7*, BBR can upregulate them more than 3 times compared with the normal control (*P* < 0.01) (Fig. 6f and h). BBR can increase the expression of *Ndufs4*, *Ndufa12*, *Ndufaf2*, *Ndufa8*, *Ndufb11* and *Uqcc2* more than 2 times compared with the normal control (*P* < 0.01, *P* < 0.05) (Fig. 6g, h and j). Meanwhile, BBR can increase the expressions of the subsets of *Mrp*, *Mrps 6*, *Mrps10* and *Mrps17,* about 2 times than the normal control (*P* < 0.05) (Fig. 6i). However, CORT did not decrease the gene expressions with difference to the proteomic results in the model group, suggesting that the proteomics results in vitro need to be validated by further experiments in vivo (Fig. 6f-j).

In addition, BBR can antagonize these CORT-mediated expression of BDNF, GR, and CREB (cAMP response element-binding protein) and increase their expressions up to normal level (Fig. 6k), suggesting the validity of this CORT-induced depressive-like behavior in mice. Moreover, after CORT treatment, proteins of NMDA receptor (NMDAR) and AMPA receptor (AMPAR), the postsynaptic membrane excitatory ion channel receptors, were downregulated 36% and 66% respectively. BBR can antagonize these CORT-mediated changes (Fig. 6l) and enhance the expression up to normal level. Notably, the discrepancy between the mRNA and protein level of NMDA receptor (NMDAR) might be regulated via exceptional mechanism that needs further investigation. Nevertheless, the results of protein expression were in agreement with the mRNA expression results (except NMDAR), indicating that the mice with CORT-induced depressive behavior presented the pathophysiologic changes (Fig. 6m-o).

## Discussion

In the observation of mouse neural stem cells in vitro, we found that CORT can affect the physiological and biochemical processes-related proteomic profiles of cells extensively, among which the mitochondrial oxidative respiratory chain was extensively studied. We found that the CORT affects the respiratory chain is mainly mediated by a subset of NADH-related enzymes. Chronic administration of CORT induced mice depression, which was associated with the downregulated expression of the molecular biomarkers of depression, such as BDNF and GR. In particular, oxidative phosphorylation related function proteins were also downregulated. In addition to the correction effect of BBR on the CORT-induced depression behavior, BBR significantly up-regulated the expression of proteins that were suppressed by CORT, which suggested as the mechanism underlying the anti-depression effect of BBR.

Cultured cells are the mostly used model in previous depression-related studies, such as glioma C6 cells [50], SH-SY5Y neuroblastoma cells [51], and pheochromocytoma (PC12) cells [52,53]. In this study, we observed the effect of CORT on C17.2 cells at a noninjury dose and observed this effect from a proteomic perspective. Our results based on C17.2 cells revealed that CORT can cause extensive neuronal disorders, especially in energy metabolism in the mitochondria. PTSD is mainly due to a disturbed HPA axis and slowly released corticosteroids in the body. In this pathophysiological process, the long-term high concentration of CORT causes the inhibition in the cerebral hippocampal neurons, which in turn affects BDNF and the expression of synapse receptors, thereby leading to the reversible changes in the neural circuits and their physiological functions [54,55]. Our proteomic results showed CORT extensively regulated the expression of proteins in nerve cells, reflecting the complex of pathophysological changes in the aspect of molecular biochemistry.

In addition to the serotonin system, mitochondrial lesions and their neuronal microenvironment have rising focuses in the field. Our results showed that CORT affects neuronal function by disturbing the protein expressions involving the subset of NADH dehydrogenase (ubiquinone) (*Nduf*), ubiquinol-cytochrome c reductase complex (*Uqcc*) and mitochondrial ribosomal protein (*Mrp*), thereby significantly regulate the function of mitochondrial energy metabolism and disturb the physiological activities of neurons.

*Nduf* is expressed in multiple sites on the mitochondrial oxidative respiratory chain which is involved in the electron transport of the mitochondrial oxidative respiratory chain, directly affecting the oxidative phosphorylation process. The downregulation of the expression of NADH dehydrogenase proteins induced by CORT disorders the normal physiological functions of the respiratory chain, affecting the energy supply and the respiration of cells. *Uqcc* mainly regulates the respiratory chain 3 complex. UQCC2, the subset of UQCC, acts directly on the oxidized respiratory chain III complex, influencing the oxidative energy supply of mitochondria [56]. CORT can downregulate UQCC2 expression, which could directly disturb the oxidative phosphorylation process of cells and change the physiological functions of neurons. Moreover, a relationship of network regulation among *Ndufs* and *Uqcc* could affect mitochondrial respiratory chain function as well as energy metabolism in synergy. MRP is an important nuclear protein in mitochondria, correlating a variety of mitochondrial functions. Components of the translational system, including the mitochondrial ribosomal proteins, are encoded by the nuclear genome and subsequently translocated into the mitochondria. Mutations in *Mrp* genes that reduce gene expression have a conserved life-extending effect in both mice and *C. elegans* [57,58]. Recent studies have found that *Mrp* is associated with cognitive function [59]. CORT can decrease *Mrp* expression, suggesting CORT may be involved in the impairment of anxiety and depression and related cognitive functions.

As mentioned above, CORT can suppress the protein expressions correlating to energy metabolism in mitochondria, and BBR can reverse this down-regulation, suggesting BBR can antagonize the changes of CORT-induced depression. This finding suggests the importance of mitochondrial oxidative respiration in the pathophysiological process of depression. Previous studies reported the inhibitory effect of BBR on mitochondria [60,61]. However, our results firstly showed that BBR can antagonize the suppression of the protein expression caused by CORT, in particular the expression of the subset of proteins of mitochondrial oxidative respiratory chain.

Stress, such as PTSD, can cause chronic high level of GC and in turn damage the neurons of the brain. Stress-like forced swimming can induce the down-regulation of GR expression in the hippocampus of mice [62], showing the relationship among stress, GC and GR expression. Our results showed CORT can cause the negative feedback inhibition of GR in nerve cells, which in turn influences neural function because GR is mainly related to cell growth, cell survival, and BDNF expression in hippocampal neurons [63]. According to data analysis, the GR gene *Nr3c1* can act on the BDNF1 transcriptional promoter region to promote the expression of BDNF1 (https://epd.epfl.ch/cgi-bin/get_doc?db=mmEpdNew&format=genome&entry=Bdnf_1). Therefore, the down-regulation of GR expression can directly affect the physiological function of nerve cells [64], although it does not cause neural necrotic apoptosis or the inflammatory response at a safety concentration.

In the present study, the body weight of the mice decreased significantly after CORT administration, BBR can ameliorate the body-weight decline suggesting the role of BBR against CORT. The sucrose preference experiment often shows the psychiatric state of mice with anxiety and depression induced by CORT [65]. Our results presented that the sucrose-water consumption of CORT mice was significantly lower than that of the normal control, implying a certain depression of mice. After BBR treatment, the sucrose-water consumption of the mice was significantly increased to the normal level, meaning the depression was ameliorated by BBR. Simultaneously, the urine volume was also increased along with the drinking water increase, suggesting the normal renal function and the balance between income and outcome. In terms of cerebral neurons, BBR can increase the expression of BDNF, CREB, GR, AMPAR and NMDAR, suggesting BBR antidepressant effect on the neuronal dysfunction caused by CORT [66]. Moreover, BBR can simultaneously reverse the down-regulation of mitochondrial oxidative phosphorylation-related proteins causing to produce an antagonistic effect and ensuring the energy supply and physiological activity of neurons.

We conducted in-depth systematic research on the molecular mechanism of BBR action and found that the target of BBR is the two aspects of gene expression (TATA-box in gene transcription) and mRNA stability (poly A in protein translation) [67–70]. Due to the extensive nature of these targets, BBR exhibits a broad and complex role at the protein level. The diversity of BBR targets for neuronal cells in the depressive-like behaviors of CORT is also the initial target for its role in DNA or RNA. Thus, further studies on the mechanisms of BBR anti-depression will be helpful, which is also the direction for further research in the future.

Mitochondria are the unit of cellular energy. The ATP produced in mitochondria provides energy for the physiological activities of neural cells. The production of ATP through oxidative phosphorylation is a key method by which mitochondria provide energy to the cell [71]. Thereby, ATP produced by the mitochondria decreases under stress while oxygen free radicals increase, affecting the energy supply in the physiological activities of neural cells, and initiates the apoptosis due to releasing cytochrome C and activating Caspase pathway, resulting in the nerve cell disorder [72–74], being supported by the experimental data of cerebral energy metabolism disorder on clinical depression cases and experimental depression animals [75–77]. Therefore, mitochondrial energy metabolism is of importance in the pathogenesis of depression.

Taken together, our results indicate that CORT-induced depression is associated with the inhibitory oxidative phosphorylation in mitochondria. BBR can antagonize the effect of CORT on mice behavior, as well as the expression of proteins that involve in mitochondrial oxidative phosphorylation processes. Our study provides new mechanism underlying the anti-depression effect of BBR.

## Data Availability

The data generated or analyzed during this study are included in this published article.
